# Metabolomics reveals metabolic changes in male reproductive cells exposed to thirdhand smoke

**DOI:** 10.1038/srep15512

**Published:** 2015-10-22

**Authors:** Bo Xu, Minjian Chen, Mengmeng Yao, Xiaoli Ji, Zhilei Mao, Wei Tang, Shanlei Qiao, Suzaynn F. Schick, Jian-Hua Mao, Bo Hang, Yankai Xia

**Affiliations:** 1 State Key Laboratory of Reproductive Medicine, Institute of Toxicology, Nanjing Medical University, Nanjing 211166, China; 2 Key Laboratory of Modern Toxicology of Ministry of Education, School of Public Health, Nanjing Medical University, Nanjing 211166, China; 3 Department of Endocrinology, Jiangsu Province Official Hospital, Nanjing Medical University, Nanjing 210024, China; 4 Department of Medicine, Division of Occupational and Environmental Medicine, University of California, San Francisco, CA 94143, USA; 5 Department of Cancer & DNA Damage Responses, Life Sciences Division, Lawrence Berkeley National Laboratory, Berkeley, CA 94720, USA

## Abstract

Thirdhand smoke (THS) is a new term for the toxins in cigarette smoke that linger in the environment long after the cigarettes are extinguished. The effects of THS exposure on male reproduction have not yet been studied. In this study, metabolic changes in male germ cell lines (GC-2 and TM-4) were analyzed after THS treatment for 24 h. THS-loaded chromatography paper samples were generated in a laboratory chamber system and extracted in DMEM. At a paper: DMEM ratio of 50 μg/ml, cell viability in both cell lines was normal, as measured by the MTT assay and markers of cytotoxicity, cell cycle, apoptosis and ROS production were normal as measured by quantitative immunofluorescence. Metabolomic analysis was performed on methanol extracts of GC-2 and TM-4 cells. Glutathione metabolism in GC-2 cells, and nucleic acid and ammonia metabolism in TM-4 cells, was changed significantly by THS treatment. RT-PCR analyses of mRNA for enzyme genes Gss and Ggt in GC-2 cells, and TK, SMS and Glna in TM-4 cells reinforced these findings, showing changes in the levels of enzymes involved in the relevant pathways. In conclusion, exposure to THS at very low concentrations caused distinct metabolic changes in two different types of male reproductive cell lines.

During the past few years, thirdhand smoke (THS) has emerged as a new idea in the field of tobacco pollution and control. THS is defined as residual tobacco smoke pollutants that 1) remain on surfaces or in dust after tobacco has been smoked, 2) are re-emitted into the gas phase, and/or 3) react with other compounds in the environment to produce secondary pollutants^1^,^2^. THS exposure can result in involuntary ingestion, inhalation, or dermal contact with pollutants in dust, in the air, and on carpets, walls, furniture, clothing, hair or skin of smokers^1^. Infants and children are 100 times more sensitive than adults to house dust^3^ because of their immature respiratory, immune and metabolic systems. Their behaviors (crawling, sucking, hand-to-mouth ingesting) also increase their susceptibility to THS. THS in dust, air or on surfaces degrades slowly indoors, and can persist for months^4^. Non-smokers living in former smoker houses which were vacant a median of 62 days are exposed to THS in dust and on surfaces^5^. Because of its persistence, THS has the potential to increase the duration of exposure to smoke toxins.

Recent research has shown that THS can cause significant cellular changes at realistic concentrations. In 2013, Hang *et al.* demonstrated for the first time that exposure to THS generated in laboratory chamber systems caused significant DNA damage in cultured human cell lines^6^. In 2014, Martins-Green *et al.* reported that THS exposure resulted in damage to multiple organs and behavioral alterations in mice^7^. A population-based study from South Korea showed an increase of prevalence of respiratory symptoms in children exposed to THS due to parental smoking^8^. These data have provided preliminary evidence suggesting that THS exposure can induce health effects.

No one has yet studied the effects of THS exposure on the reproductive system. Therefore, in the present study, we investigated the effects of THS exposure on metabolism in two male reproductive cell lines, GC-2 and TM-4. The GC-2 cell line was originally derived from immortalized murine spermatogonia, and the TM-4 cell line was derived from murine sertoli cells (the main components of the blood-testis barrier). Spermatogonia are undifferentiated male germ cells. Sertoli cells provide both structural and nutritional support to germ cells, and maintain the spermatogenous microenvironment during spermatogenesis. Both cell lines are useful models in testing the effects of chemicals on male reproductive system, including the study of metabolomics^9^,^10^,^11^.

Metabolomics is a recently developed approach for detecting dynamic variations in the quantity and identity of small molecules, which are low molecular weight (<900 daltons) organic compounds that may help regulate a biological process^12^. It is useful for assessing changes in multiple biochemical pathways after exposure to chemicals and elucidating the mechanisms linking exposure to disease processes^13^. Our previous studies have demonstrated that metabolomics is a promising tool in studying reproductive toxicity^10^,^11^.

In this study, to test the effects of THS exposure on male reproductive cells, metabolomics analysis and Reverse Transcription-Polymerase Chain Reaction (RT-PCR) were conducted in THS-treated GC-2 and TM-4 cells. Results showed that low concentrations of THS in which no significant effects of THS on general cytotoxicity and multi-parameter cytotoxicity could alter metabolism in GC-2 and TM-4 cells, indicating potential effects of THS on male reproduction.

## Methods

### Cell culture and THS treatment

Smoke samples were generated in a laboratory system to simulate chronic THS pollution, as described previously^6^,^14^. Briefly, the chronic THS samples were generated by exposing chromatography paper (3 MM Chr, Whatman, GE Lifesciences, Pittsburgh, PA, USA) to cigarette smoke in a 6 m^3^ stainless steel chamber for a total of 32 h over 20 days, with a total smoke exposure of 545 mg particulate material.

THS-exposed paper was weighed and cut into small pieces. The tobacco chemicals were extracted at a ratio of 0.15 g paper to 3 ml serum-free Dulbecco’s Modified Eagle’s Medium (DMEM) as described previously^6^. Extracts were stored at 4^o^ C in the dark. A serial dilution of the stock solution with DMEM was prepared to equal 0.05, 0.5, 5, 50, 500, 5000 and 50,000 μg paper per ml (μg/ml). The concentrations of nicotine, cotinine and 4-methylnitrosamino-1-(3-pyridyl)- 1-butanone (NNK) in the THS extracts and control DMEM were measured using Ultimate 3000 UHPLC system (Dionex) and Q-Exactive quadrupole-obitrap mass Spectrometer (Thermo Fisher Scientific). Nicotine-D3 was purchased from CDN Isotopes (Quebec, Canada). Nicotine, cotinine and NNK were purchased from Dr. Ehrenstorfer GmbH (Germany). The mobile phase consisted of 0.1% formic acid in acetonitrile and water (95:5) with 10 mM ammonium acetate (solvent A) and 0.1% formic acid in water (solvent B). The injections were loaded onto a HILIC column (1.7 μm, 2.1 × 100 mm), and gradient was used for elution.

GC-2 spd(ts) (ATCC # CRL-2196) and TM-4 (ATCC # CRL -1715) cells were purchased from ATCC (Manassas, VA, USA) and cultured in DMEM containing 10% Fetal bovine serum (FBS), 100 U/mL penicillin and 100 μg/mL streptomycin at 37 °C and 5% CO_2_, then diluted to the desired concentrations in serum-free medium before use.

### Cell viability assay

Cell viability was evaluated by the MTT assay. 5 × 10^4^ cells per well were plated in 96-well plates. After exposure to THS at different concentrations, 25 μl of MTT (5 mg/ml) was added, then the cells were incubated for 4 h at 37 °C, and the absorbance was determined at 490 nm.

### Cell cycle and apoptosis assay

To determine if THS could affect the cell cycle and apoptosis of GC-2 and TM-4 cells, flow cytometric analysis was used. Cells were seeded at ~1 × 10^6^ cells per well in 6-well plates. After exposure to THS (50 μg/ml) serum-free medium, cells were washed with phosphate buffer saline (PBS) and harvested with trypsin after 24 h treatment. The cell cycle and apoptosis were analyzed by FACS Calibur Flow Cytometry (BD Biosciences, NJ, USA) as described in previous study^10^.

### Multi-parameter cytotoxicity analysis by high content screening

Multi-parameter cytotoxicity was analyzed by cell-based high-content screening (HCS) analysis (Thermo Scientific Cellomics ®ArrayScan® VTI HCS Reader, Pittsburgh, USA) as described previously^10^. Briefly, cells were plated at a density of 5 × 10^4^ cells/ml in Collagen I-coated 96-well plates (BD Biocoat® Plates) and incubated for 24 h with 50 μg/ml THS, control, or 120 μM valinomycin as positive control. Then, the cells were fixed and stained using the Multiparameter Cytotoxicity 3 Kit (Cellomics) which tests nuclear integrity, cell membrane integrity; cytochrome c levels and mitochondrial membrane potential. A 20x objective was used to collect images. For each treatment, four independent wells were examined, and 16 fields per well were captured for analysis.

### Metabolomic analysis

GC-2 and TM-4 cells at 80% confluency in 10 cm dishes were exposed to 50 μg/ml THS for 24 h. The metabolites were extracted from cells using 50% methanol, and gas chromatography-time-of-flight mass spectrometry (GC-TOF MS) metabolomics analysis was performed using an Agilent 7890 gas chromatography system coupled with a Pegasus 4D time-of-flight mass spectrometer. The details of sample preparation and metabolomic analysis can be found in our previous study^10^.

### RT-PCR assay

Total RNA was isolated using TRIZOL reagent (Invitrogen, Carlsbad, CA). cDNA syntheses was performed with 1 μg of total RNA (Takara, Tokyo, Japan). The mRNA levels of metabolic enzyme genes were analyzed using SYBR PCR Master Mix reagent kits (Takara), which include glutathione synthetase (Gss), gamma-glutamyltransferase (Ggt), thymidine kinase (TK), spermine synthase (SMS), and glutamate-ammonia ligase (Glna). The primers are shown in Table S1. All real-time PCR reactions were carried out on an ABI 7900 Fast Real-Time System (Applied Biosystems, CA, USA). All RT-PCR experiments were repeated at least three times.

### Analysis of the reactive oxygen species (ROS) content

To quantify the effect of THS exposure on intracellular ROS in GC-2 cells, flow cytometric analysis was used to detect the oxidation sensitive probe DCFH-DA. GC-2 cells were incubated overnight and subsequently exposed to THS (50 μg/ml) or control medium for 24 h. The cellular fluorescence intensity was measured after 30 min incubation with 5 μM DCFH-DA at 37 °C, followed by FACS Calibur Flow Cytometry (BD Biosciences, NJ, USA).

### Data analysis

HCS analysis was performed using the Automated Image and Data Analysis software. Statistically significant differences between the treatments and the control were determined by one-way ANOVA, followed by Dunnett’s multiple comparison test. Statistical analysis was performed and presented with GraphPad prism 5 software. All tests of statistical significance were two-tailed, and the statistical significance was set at *p* < *0.05*. Significant differences of metabolites between treatment and control were tested using t-test analysis. The Orthogonal Partial Least Squares-Discriminant Analysis (OPLS-DA) model based on metabolomics data was established using SIMCA-P 13.0 (Umetrics, Umea, Sweden).

## Results

### Effects of THS on cell viability and toxicity

**Figure 1:**
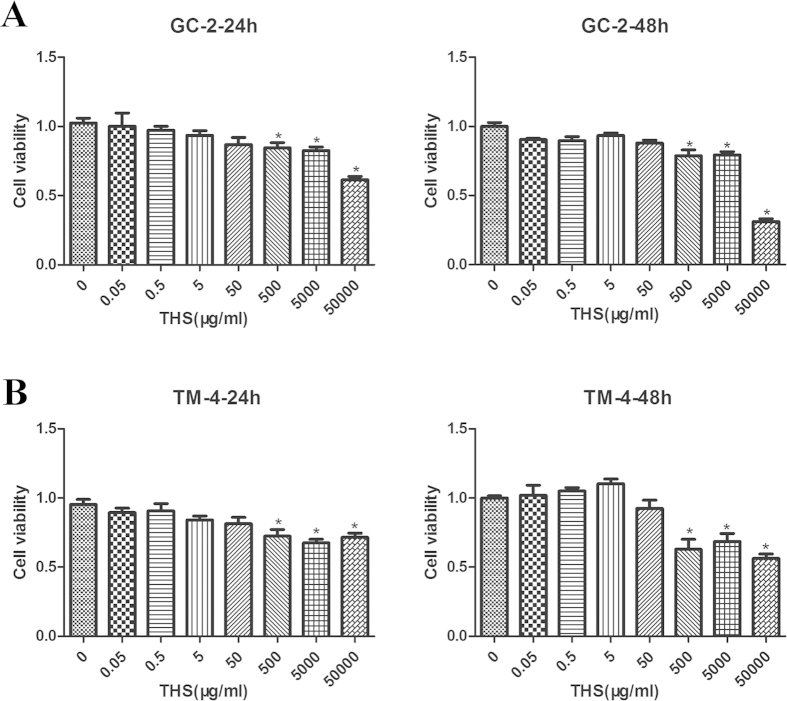
Dose response of GC-2 (A) and TM-4 (B) cells to THS exposure in cell viability at 24 or 48 h. Cells were exposed to various concentrations of THS (0 to 50000 μg/ml) in DMEM and MTT assay was used to analyze cell viability.

We first investigated the effects of THS on cell viability and cytotoxicity. GC-2 and TM-4 cells were exposed to serial dilutions of THS DMEM extract, from 0 to 50,000 μg/ml, for 24 and 48 h. Dilutions from 0 to 50 μg/ml THS did not affect cell viability in either GC-2 or TM-4 cells, while 500 to 50,000 μg/ml significantly reduced viability (Fig. 1A,B). Therefore, in all of the following experiments, cells were exposed to THS at 50 μg/ml for 24 h, a non-cytotoxic dose. At this dilution, the concentrations of nicotine, cotinine and NNK were 84.0 pg/ml, 7.3 pg/ml and 0.16 pg/ml, respectively.

**Figure 2:**
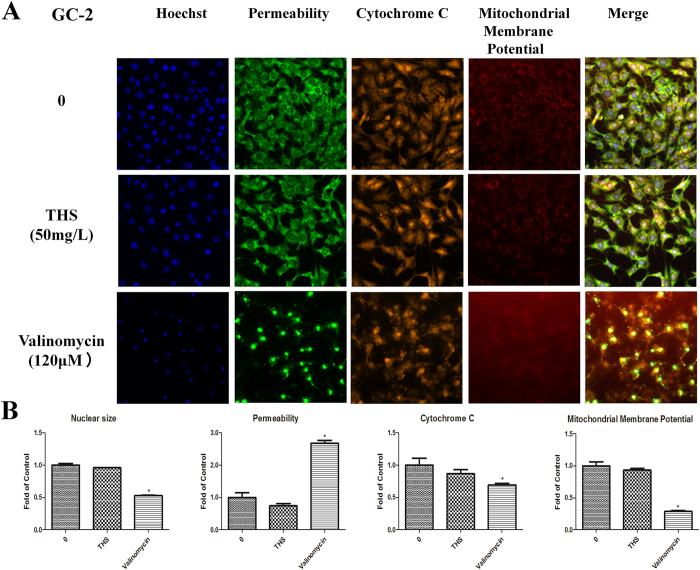
Representative images from the high-content screen after THS exposure in GC-2 cells. (**A**) Staining for nuclei (blue), cell membrane permeability (green), cytochrome c (yellow) and mitochondria membrane potential (red). Images were acquired with the ArrayScan HCS Reader with a 20x objective. (**B**) The relative expressions of nuclear size, permeability, cytochrome c and mitochondria membrane potential. *indicates significant difference when the values were compared to that of the control (*p* < *0.05*).

**Figure 3:**
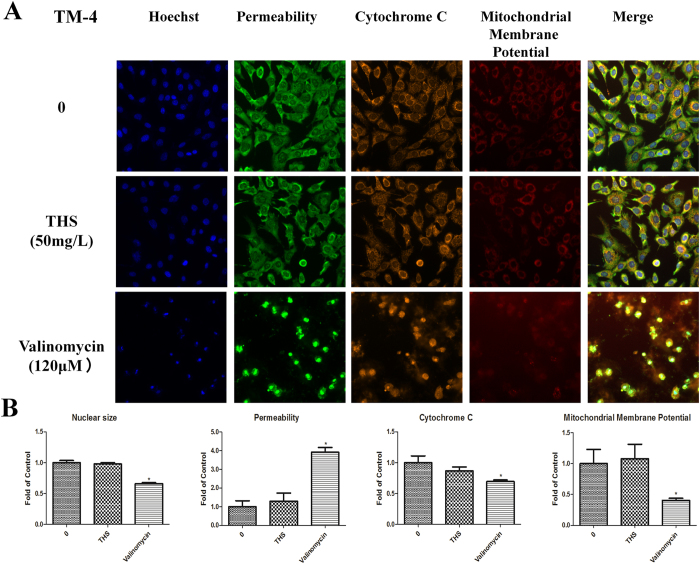
Representative images from the high-content screen after THS exposure in TM-4 cells. (**A**) Staining for nuclei (blue), cell membrane permeability (green), cytochrome c (yellow) and mitochondria membrane potential (red). Images were acquired with the ArrayScan HCS Reader with a 20x objective. (**B**) The relative expressions of nuclear size, permeability, cytochrome c and mitochondria membrane potential. * indicates significant difference when the values were compared to that of the control (*p* < *0.05*).

Cell cycle, apoptosis, and HCS multi-parameter cytotoxicity analyses carried out with 50 μg/ml THS extract sample for 24 h showed no significant differences between treated and control groups (Fig. S1, Fig. 2 and Fig. 3). These findings are consistent with the lack of effects on cell viability by the MTT assay.

### Metabolic changes after THS exposure in GC-2 cells

**Figure 4:**
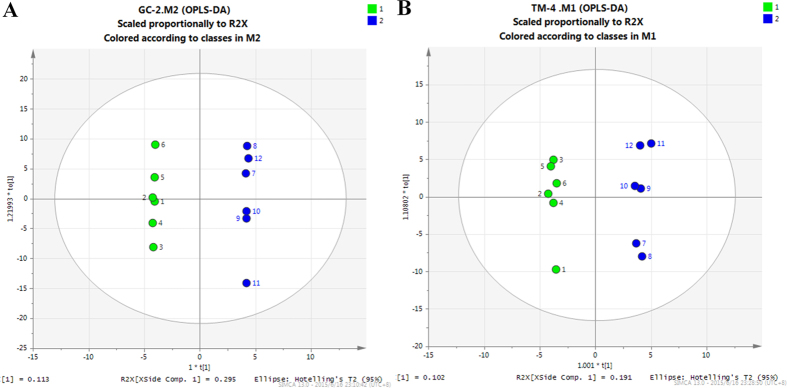
OPLS-DA score plots for GC/MS data. The green circle represents the control group, and the blue circle represents the THS treatment group. (**A**) OPLS-DA score plot showed good discrimination between the THS treatment and control groups in GC-2 cells. (**B**) OPLS-DA score plot showed good discrimination between the THS treatment and control groups in TM-4 cells.

**Figure 5:**
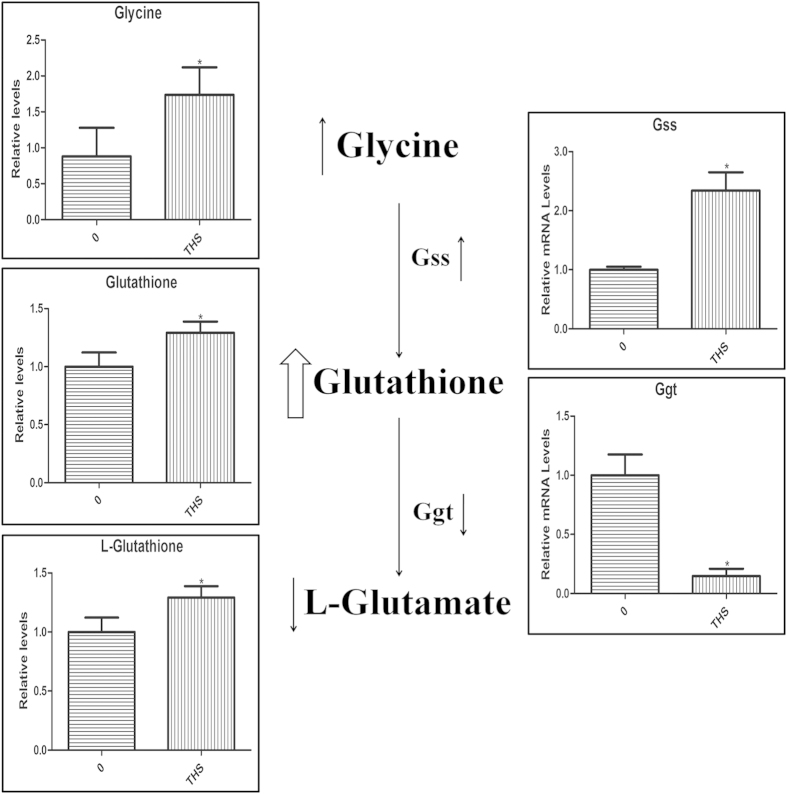
The relative levels of metabolites and metabolic enzyme gene mRNAs in GC-2 cells after THS exposure. The left panel shows the GC-MS results of levels of metabolites, glycine, glutathione and L-glutamate, in the glutathione metabolism. The right panel exhibits the RT-PCR levels of related metabolic enzyme genes, Gss and Ggt. In the middle, the metabolic pathway of glutathione is depicted schematically. *Indicates significant difference when the values were compared to that of the control (*p* < *0.05*). All tests were performed in triplicate and presented as means ± SE.

The OPLS-DA model separated samples in treatment and control groups in GC-2 cells (Fig. 4A). Metabolic profiles were shown in Table S2, and statistically significant changed metabolites were shown in Table 1 (VIP > 1 and p < 0.05)^15^. To identify key metabolism alterations after THS exposure in GC-2 cells, we compared metabolite levels between treatment and control and analyzed metabolic pathways using Kyoto Encyclopedia of Genes and Genomes (http://www. genome.jp/kegg/). We found that the concentrations of glutathione (GSH) and glycine were significantly higher in THS-treated cells than in untreated cells. Glutathione is an important cellular antioxidant, formed by the reaction of glycine and γ-glutamylcysteine. Accordingly, the reaction product of GSH, glutamate, was decreased, suggesting increased biosynthesis and reduced catabolism of GSH (Fig. 5). Interestingly, no significant difference in ROS content was observed between the treatment and the control as examined by flow cytometry (Fig. S2). To further probe the changes in the glutathione pathway, the mRNA levels of glutathione synthetase (Gss) and gamma-glutamyltransferase (Ggt) were analyzed by qRT-PCR in GC-2 cells. Gss is the enzyme catalyzing the conversion of glycine to glutathione, while Ggt catalyzes the conversion of glutathione to glutamate. We found increased mRNA levels of Gss and decreased mRNA levels of Ggt, supporting the metabolomic findings. These changes in mRNA number support our hypothesis of increased biosynthesis and reduced catabolism of GSH after THS treatment (Fig. 5).

### Metabolic changes after THS exposure in TM-4 cells

**Figure 6:**
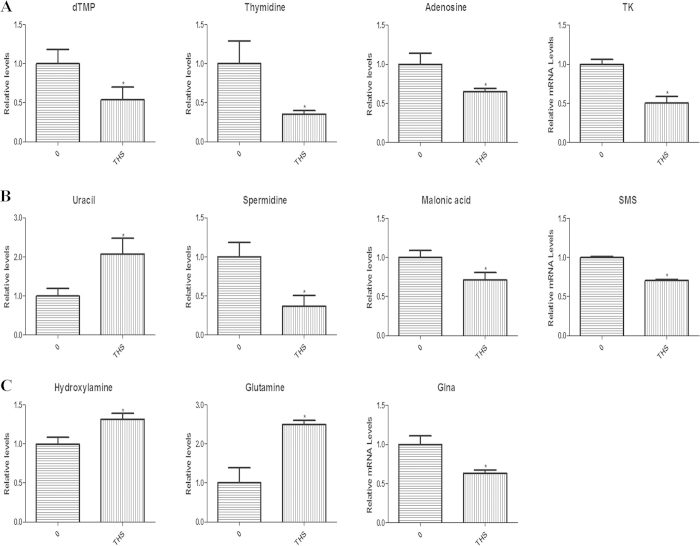
The relative levels of metabolites and metabolic enzyme gene mRNAs in TM-4 cells after THS exposure. (**A**) The GC-MS results of dTMP, Thymidine and Adenosine, and the RT-PCR result of related metabolic enzyme gene, TK; (**B**) The GC-MS results of Uracil, Spermidine and Malonic acid, and the RT-PCR result of related metabolic enzyme gene, SMS; (**C**) The GC-MS results of Hhydroxylamine and L-Glutathione, and the RT-PCR result of related metabolic enzyme gene, Glna. *Indicates significant difference when the values were compared to that of the control (*p* < *0.05*). All tests were performed in triplicate and presented as means ± SE.

The OPLS-DA model separated samples in treatment and control groups in TM-4 cells (Fig. 4B). Metabolic profiles were shown in Table S2, and statistically significant changed metabolites were shown in Table 2 (VIP > 1 and p < 0.05)^15^. Interestingly, there were many altered metabolites related to nucleic acid metabolism including uracil, thymidine, deoxythymidine monophosphate (dTMP), malonic acid, spermidine and adenosine (Fig. 6A,B). This suggests that THS could induce genetic effects in TM-4 cells. THS exposure also caused an increase in ammonia metabolism in TM-4 cells, as shown by the increase of two products of ammonia, hydroxylamine and glutamine (Fig. 6C). The mRNA levels of the relevant metabolic enzymes in these pathways were also analyzed in TM-4 cells. The mRNA levels of thymidine kinase (TK), which catalyzes the formation of dTMP through the phosphorylation of thymidine, spermine synthase (SMS), which catalyzes the transformation of spermidine to spermine, and glutamate-ammonia ligase (Glna), which catalyzes the conversion of ammonia to glutamine, all decreased significantly in THS treated cells. Both increased downstream metabolites converted from ammonia including glutamine and decreased expression of the enzyme for catalyzing this process may contribute to a significant increase of ammonia in TM-4 cells after THS treatment.

## Discussion

Exposure to secondhand smoke (SHS) causes cardiovascular effects^16^, asthma^17^, lung cancer^18^ and other long-term diseases. As a new cigarette hazard, THS has aroused increasing public attention. However, little is known about the effect of THS exposure on male reproduction.

Our metabolomic analysis provides the first evidence that THS, at low concentrations, *i.e*., 50 μg/ml paper sample, can cause distinct disturbances in the levels of metabolites and the gene expression of related metabolic enzymes in spermatogonia (GC-2) and sertoli-derived (TM-4) cells. Notably our results showed that there was no significant difference in cell viability, cell cycle, and apoptosis between THS treatment and the control at this concentration of THS. This suggests that the metabolic processes in male reproductive cells may be particularly sensitive to THS exposure.

THS exposures are characterized by lower concentrations than SHS exposures and longer duration of exposure^1^. The levels of nicotine and cotinine in the THS samples we used were 2 to 3 orders of magnitude below those seen in the plasma of active smokers^19^. Cotinine values were 1-2 orders of magnitude below those seen in the serum of nonsmokers exposed to secondhand smoke^20^. NNK is difficult to measure directly in biofluids^21^, however, a typical cigarette yields 200-400 ng of NNK to the smoker^22^ and releases 200-1,400 ng into the air as secondhand smoke^23^. NNK also forms in THS by nitrosation of nicotine^2^,^14^,^24^. Thus the relatively small difference between nicotine and NNK concentrations in the exposure medium is likely to reflect a realistic concentration ratio between these compounds in real-world THS exposures.

Glutathione metabolism was significantly changed in GC-2 cells after THS exposure, with increased levels of GSH and its upstream metabolite glycine and a decreased level of GSH’s product, glutamate. In addition, the expression of Gss was increased, while the Ggt expression was decreased. These results indicate increased biosynthesis and reduced catabolism of GSH. This may mean that the oxidants in THS caused an adaptive response in GC-2 cells. GSH plays a crucial role in ROS scavenging antioxidant defense^25^,^26^.

In line with a previous study about marijuana smoke^27^, our data suggest that GSH was involved in the antioxidant response to a smoke-related exposure. Previous studies confirmed that 24 h chemical exposures can cause an increase in ROS content^28^,^29^. The lack of detectable changes in ROS concentrations in GC-2 cells in this study suggests that the changes in GSH, glycine, glutamate, Gss and Ggt are sufficient to prevent oxidative stress. However, at higher levels of THS exposure, increased ROS content might occur in the exposed cells.

Many of the metabolites changed in TM-4 cells after THS exposure, including uracil, thymidine, dTMP, malonic acid, spermidine and adenosine, are related to nucleic acid metabolism. These observations are also supported by mRNA analysis of the expression of the corresponding metabolic enzymes. Pyrimidine metabolism (dTMP, thymidine), which is involved in nearly all the biochemical processes relating to nucleic acid metabolism, was also significantly changed in TM-4 cells. Previous work indicates that the disorders of pyrimidine metabolism are associated with developmental delay^30^. A tobacco smoke, tobacco smoke condensate, second-hand smoke^31^,^32^ and THS are also proven genotoxins^6^. These subtle changes in nucleic acid metabolism may provide us new insight into the mechanisms of THS-induced genetic toxicity, especially in sertoli cells.

Two metabolic products of ammonia, including hydroxylamine and glutamine, were found to have increased levels. Meanwhile, the level of Glna was found to be decreased in THS-treated TM-4 cells, indicating the increase of ammonia in these cells. As ammonia is toxic^33^, THS may exert toxic effect by disturbing this metabolite metabolism.

The metabolic responses were various with different cell lines after THS treatment, which was similar to our previous study about metabolic responses of gold nanoparticles treated these two cell lines^10^. The differences in the metabolic responses to THS exposure in these two different cell lines from the male reproductive system may reflect distinct molecular mechanisms underlying both metabolism and function of these different cells. GC-2 cell line is useful for the study of spermatogenesis, while TM-4 cell line is the major supportive cell type of the testes. Our results showed that glutathione metabolism in GC-2 cells, and nucleic acid and ammonia metabolism in TM-4 cells, were changed significantly by THS treatment. As the changes in glutathione metabolism might be a protective response, while changes in nucleic acid and ammonia metabolism might be harmful. Therefore, THS might be more unfavorable to TM-4 cells than GC-2 cells. And further investigation is warranted.

## Conclusions

This metabolomic study shows that THS exposure at a low, non-cytotoxic dose can significantly alter metabolism in two different types of male reproductive cell lines, thus providing the first evidence that THS may have biological effects on reproductive cells at realistic concentrations. This provide new insight into the mechanisms underlying toxin-induced genetic and cytotoxicity in both germ and sertoli cells and the potential health risks of THS exposure.

## Additional Information

**How to cite this article**: Xu, B. *et al.* Metabolomics reveals metabolic changes in male reproductive cells exposed to thirdhand smoke. *Sci. Rep.* **5**, 15512; doi: 10.1038/srep15512 (2015).

## Supplementary Material

Supplementary Information

## Figures and Tables

**Table 1:** Statistically significant changed metabolites in GC-2 cells.

Metabolite	VIP	P Value	Fold change
21-Hydroxypregnenolone	1.832	0.050	1.686
2-Ketoadipate	1.684	0.044	1.377
4-Vinylphenol dimer	0.553	0.028	1.133
5-Aminovaleric acid	1.131	0.013	1.428
9-Fluorenone	1.858	0.026	1.607
Cytidine-monophosphate	1.458	0.020	1.656
Diglycerol	1.168	0.013	0.541
Ethanolamine	1.271	0.027	1.224
Glutathione	1.489	0.046	1.292
Glycine	1.542	0.039	1.891
Hydrocortisone	0.358	0.006	0.889
L-glutamate	1.959	0.036	0.436
Oxalic acid	1.705	0.026	1.341
Oxoproline	1.477	0.033	1.215
Pantothenic acid	1.899	0.016	1.404
P-benzoquinone	0.855	0.008	1.143
Pelargonic acid	1.334	0.043	1.107
Synephrine	1.570	0.013	1.385

**Table 2:** Statistically significant changed metabolites in TM-4 cells.

Metabolite	VIP	P Value	Fold change
Adenosine	1.781	0.036	0.650
Cis-gondoic acid	1.035	0.027	1.339
Creatine	2.263	0.048	2.001
dTMP	1.500	0.030	0.649
Glutamine	2.424	0.007	2.495
Hydrocortisone	1.035	0.018	0.522
Hydroxylamine	2.040	0.043	1.317
Indolelactate	1.551	0.044	0.545
Malonic acid	1.793	0.033	0.712
N(epsilon)-Trimethyllysine	0.860	0.043	0.792
N-alpha-Acetyl-L-ornithine	1.092	0.032	1.282
Naphthalene	2.004	0.030	1.303
Nicotinic acid	1.048	0.026	1.430
Noradrenaline	2.424	0.027	1.424
O-methylthreonine	0.376	0.026	0.873
Pantothenic acid	1.717	0.024	1.200
Spermidine	2.039	0.036	0.367
Thymidine	1.770	0.020	0.356
Uracil	2.048	0.019	2.074
