# Corrigendum: Metabolomics reveals metabolic changes in male reproductive cells exposed to thirdhand smoke

**DOI:** 10.1038/srep23849

**Published:** 2016-04-13

**Authors:** Bo Xu, Minjian Chen, Mengmeng Yao, Xiaoli Ji, Zhilei Mao, Wei Tang, Shanlei Qiao, Suzaynn F. Schick, Jian-Hua Mao, Bo Hang, Yankai Xia

Scientific Reports 5: Article number: 1551210.1038/srep15512; published online: 10222015; updated: 04132016

The Acknowledgements section in this Article is incomplete.

“This study was supported by National 973 Program (2012CBA01305); National Science Fund for Outstanding Young Scholars (81322039); National Natural Science Foundation (31371524); Distinguished Young Scholars of Jiangsu Province (BK20130041); Priority Academic Program Development of Jiangsu Higher Education Institutions (PAPD); New Century Excellent Talents in University (NCET-13-0870) and the California Tobacco-Related Disease Research Program (TRDRP) Grant 19XT-0070 (to B.H.) under U.S. Department of Energy (Contract no. DE-AC02-05CH11231).”

should read:

“This study was supported by National 973 Program (2012CBA01305); National Science Fund for Outstanding Young Scholars (81322039); National Natural Science Foundation (31371524); Distinguished Young Scholars of Jiangsu Province (BK20130041); Priority Academic Program Development of Jiangsu Higher Education Institutions (PAPD); New Century Excellent Talents in University (NCET-13-0870) and the California Tobacco-Related Disease Research Program (TRDRP) Grant 19XT-0070 (to B.H.) under U.S. Department of Energy (Contract no. DE-AC02-05CH11231); The California Consortium on Thirdhand Smoke, California Tobacco-Related Disease Research Program (trdrp.org) grant 20PT-0184 and California Tobacco-Related Disease Research Program grant 21 ST-011).”

