# Supplementary material for: Metabolomics reveals metabolic changes in male reproductive cells exposed to thirdhand smoke
Source: Sci Rep. 2015 Oct 22;5:15512. doi: 10.1038/srep15512 (PMC4614949; doi:10.1038/srep15512)
Supplement: Supplementary Information [file srep15512-s1.pdf]

Metabolomics reveals metabolic changes in male reproductive cells exposed to thirdhand smoke

Bo Xu[a, b, 1], Minjian Chen[a, b, 1], Mengmeng Yao[a, b, 1], Xiaoli Ji[a, b], Zhilei Mao[a, b], Wei Tang[c], Shanlei Qiao[a, b], Suzaynn F. Schick[d], Jian-Hua Mao[e], Bo Hang[e*], Yankai Xia[a, b*]

[a]State Key Laboratory of Reproductive Medicine, Institute of Toxicology, Nanjing Medical University, Nanjing 211166, China

[b]Key Laboratory of Modern Toxicology of Ministry of Education, School of Public Health, Nanjing Medical University, Nanjing 211166, China

[c]Department of Endocrinology, The Affiliated Jiangyin Hospital of Wuxi Clinical School of Medicine, Nanjing Medical University, Jiangyin 214400, China

[d]Department of Medicine, Division of Occupational and Environmental Medicine, University of California, San Francisco, CA 94143, USA

[e]Department of Cancer & DNA Damage Responses, Life Sciences Division, Lawrence Berkeley National Laboratory, Berkeley, CA 94720, USA

[1]These authors contributed equally to this work.

*To whom correspondence should be addressed:

Yankai Xia, Ph.D.

State Key Laboratory of Reproductive Medicine, Institute of Toxicology,

Nanjing Medical University, 101 Longmian boulevard, Nanjing 211166, China.

Tel: +86-25-86868425, Fax: +86-25-86868427, E-mail: yankaixia@njmu.edu.cn

Bo Hang, Ph.D.

Life Sciences Division, Lawrence Berkeley National Laboratory, Berkeley, USA

Tel: +510-435-2434, Fax: 510-486-6488, E-mail: Bo_hang@lbl.gov

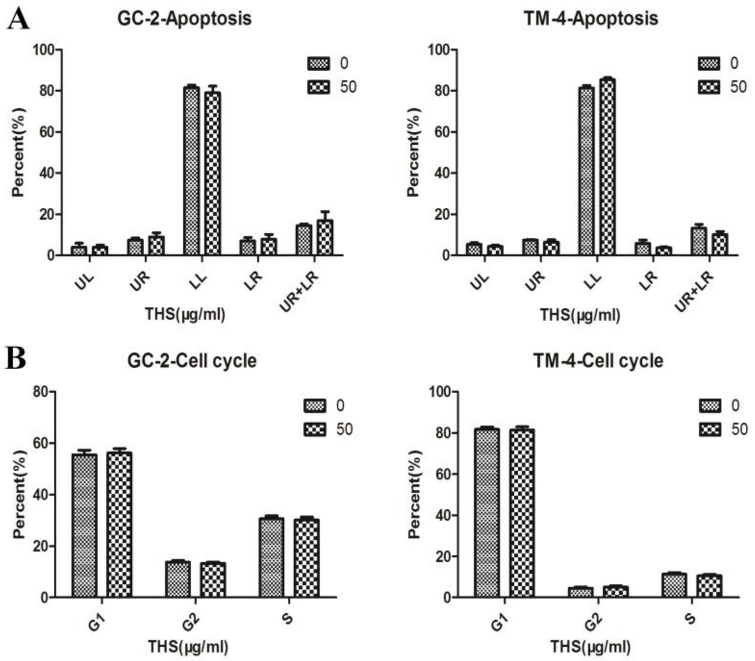

Figure S1. Effects of THS on apoptosis and cell cycle in GC-2 and TM4 cells. (A) The percentage of apoptotic cells in GC-2 and TM4 were presented in histogram. Cells in the LL quadrant indicated that they were live cells. Cells in the LR quadrant were in the early stages of apoptosis. Cells in the UR quadrant were late apoptotic. Cells in the UL quadrant indicated that they were dead cells. (B) The quantitated results of cell cycle in GC-2 and TM4 cells. Data of the experiment was expressed as a percentage of total cells.Each data point was represented as the means ± S.E. from three separate experiments in which treatments were performed in triplicate.

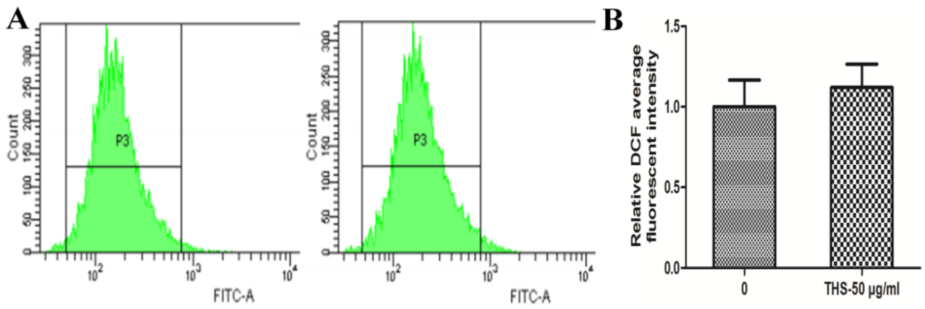

Figure S2. Comparison of ROS content in THS (50μg/ml) and control group. (A) Flow cytometry results of ROS content. x-axis, DCFH-DA fluorescence; y-axis, number of GC-2 cells. (B) The DCF average fluorescent intensity was also presented in histogram. Data were shown as the means ± SE. * indicates significant difference when the values were compared to that of the control ($p < 0.05$).

Table S1. Sequences of primers for RT-PCR.

gene		Sequences
Gss	Forward Primer	5’- CAAAGCAGGCCATAGACAGGG-3’
	Reverse Primer	5’- AAAAGCGTGAATGGGGCATAC-3’
Ggt	Forward Primer	5’- CGCAAGCCTGCTGTGTATG-3’
	Reverse Primer	5’- CGGGCATTGATAACCTCAACTT-3’
TK	Forward Primer	5’- AGTGCCTGGTCATCAAGTATGC-3’
	Reverse Primer	5’- GCTGCCACAATTACTGTCTTGC-3’
SMS	Forward Primer	5’- GCGATTACCACCCATAGTTCG-3’
	Reverse Primer	5’- TCTGCCAAATTAACATCCCCG-3’
Glna	Forward Primer	5’- CAGAGACCAACTTGAGGCACA-3’
	Reverse Primer	5’- CTCCATTCCAAACCAGGGGT-3’
GAPDH	Forward Primer	5’-AGGTCGGTGTGAACGGATTTG-3’
	Reverse Primer	5’-GGGGTCGTTGATGGCAACA-3’

Table S2. Metabolic profiles of metabolites in GC-2 cells

Metabolite	VIP	P Value	Fold change
Hydrocortisone	0.358	0.006	0.889
P-benzoquinone	0.855	0.008	1.143
5-Aminovaleric acid	1.131	0.013	1.428
Synephrine	1.570	0.013	1.385
Diglycerol	1.168	0.013	0.541
Pantothenic acid	1.899	0.016	1.404
Cytidine-monophosphate	1.458	0.020	1.656
Oxalic acid	1.705	0.026	1.341
9-Fluorenone	1.858	0.026	1.607
Ethanolamine	1.271	0.027	1.224
4-Vinylphenol dimer	0.553	0.028	1.133
Oxoproline	1.477	0.033	1.215
L-glutamate	1.959	0.036	0.436
Glycine	1.542	0.039	1.891
Pelargonic acid	1.334	0.043	1.107
2-Ketoadipate	1.684	0.044	1.377
Glutathione	1.489	0.046	1.292
21-Hydroxypregnenolone	1.832	0.050	1.686
N-Ethylglycine 1	0.978	0.058	0.533
Malonamide	0.977	0.059	1.307
Threitol	1.448	0.060	1.481
Cumic Acid	0.177	0.063	0.964
N-(3-aminopropyl)-morpholine	2.747	0.064	1.225
Methylmalonic acid	1.555	0.068	1.229
3-Methylthiopropylamine	0.939	0.071	0.690
Beta-Mannosylglycerate	1.537	0.073	0.394
Dihydroxyacetone	0.216	0.077	0.904
Thymol	1.159	0.078	1.237
(-)-Dihydrocarveol	0.596	0.085	0.728
Hypoxanthine	1.863	0.087	2.661
Pyrophosphate	0.553	0.096	0.716
Sorbitol	0.939	0.098	0.815
Glutamine	1.256	0.100	1.301
5-Methoxytryptamine	0.291	0.104	1.073
N-alpha-Acetyl-L-ornithine	1.694	0.108	1.684
Linoleic acid	2.814	0.111	1.469
Ribitol	0.076	0.113	0.980
Phenylethylamine	1.194	0.117	1.231
1,3-Diaminopropane	1.030	0.118	1.203
2'-Deoxyguanosine	1.561	0.119	0.579
Uracil	0.554	0.121	0.896

Tyrosine	0.623	0.122	1.232
Citric acid	1.745	0.122	1.587
Maleimide	0.990	0.122	1.202
N-Methyl-L-glutamic acid	0.686	0.130	0.770
D-Glyceric acid	1.784	0.130	0.615
Dithioerythritol	1.357	0.131	1.200
3-Ureidopropionate	2.046	0.133	0.741
2-Hydroxypyridine	1.191	0.137	1.261
Galactinol	1.429	0.138	1.737
5-Dihydrocortisol	2.071	0.138	1.472
3,4-Dihydroxymandelic acid	1.153	0.139	1.297
L-homoserine	0.838	0.145	1.274
3,5-Dihydroxyphenylglycine	0.994	0.147	1.136
Glycocyamine	0.895	0.151	1.407
Fucose	0.954	0.153	1.175
Fumaric acid	0.838	0.159	1.191
Pentadecanoic acid	1.118	0.159	1.298
D-erythronolactone	0.907	0.164	1.515
Oxamic acid	0.371	0.165	0.996
N-Methyl-DL-alanine	0.735	0.184	1.120
Hydrocinnamic acid	0.634	0.184	1.348
Glucose	0.179	0.185	0.915
N-epsilon-Acetyl-L-lysine	0.960	0.185	1.796
Methyl Phosphate	1.129	0.212	0.566
Tartronic acid	1.046	0.220	1.214
Alanine	0.652	0.224	0.867
Carnitine	0.951	0.231	0.649
3,6-Anhydro-D-galactose	0.965	0.231	0.838
Phosphomycin	1.392	0.234	0.552
Methyl heptadecanoate	1.103	0.240	1.437
4-Aminobutyric acid	0.918	0.247	1.129
L-Malic acid	1.204	0.271	2.035
Myristic Acid	1.150	0.275	1.565
Myo-inositol	0.166	0.276	0.946
Noradrenaline	0.709	0.282	1.220
Thymidine	0.679	0.293	1.237
Conduritol b epoxide	1.152	0.295	1.131
Adenosine	1.065	0.299	1.601
2,3-Dihydroxypyridine	0.830	0.309	0.998
Guanosine-5'-monophosphate	0.780	0.311	1.087
2-Ketobutyric acid	0.949	0.311	0.786
Elaidic acid	0.351	0.314	1.061
Aminomalonic acid	0.142	0.315	0.969
Valine	1.085	0.323	0.766

Serine	0.729	0.324	0.778
Biuret	0.608	0.327	0.922
Spermidine	0.872	0.331	1.373
Zymosterol	1.069	0.332	1.387
D-(glycerol 1-phosphate)	0.594	0.337	0.804
Methyl Palmitoleate	1.491	0.338	1.480
Ornithine	0.520	0.341	0.922
Putrescine	0.217	0.347	0.990
O-Phosphorylethanolamine	1.010	0.352	0.535
Tricetin	0.724	0.353	1.122
4-Acetamidobutyric acid	1.040	0.362	0.503
Prostaglandin E2	1.006	0.365	1.516
3-Hydroxypyridine	0.465	0.374	1.077
7-Hydroxy-4-androstene-3,17-dione	0.785	0.374	1.142
Taurine	0.223	0.377	0.925
O-Phosphoserine	0.971	0.377	1.162
Guanine	0.142	0.399	1.096
Phthalic acid	0.860	0.401	0.610
Cycloleucine	1.274	0.413	1.549
Methionine	0.790	0.419	0.834
Heptadecanoic acid	0.890	0.424	1.267
Isoleucine	0.791	0.439	0.810
Lactose	0.878	0.441	1.327
O-methylthreonine	0.781	0.450	0.714
L-Allothreonine	0.742	0.450	1.125
Cytosin	0.851	0.450	0.611
L-cysteine	0.588	0.463	1.215
Alpha-ketoglutaric acid	0.784	0.464	0.846
Asparagine	1.094	0.481	0.876
Aspartic acid	0.683	0.482	1.276
Phenylalanine	0.090	0.488	1.027
Hydroxylamine	0.723	0.499	0.846
2'-Deoxycytidine 5'-triphosphate	0.526	0.519	1.190
1-Methylhydantoin 2	1.555	0.528	0.561
2'-Hydroxyacetophenone	0.684	0.529	1.165
O-phosphonothreonine	0.145	0.529	1.274
Nicotinamide	0.605	0.536	1.141
Fructose	0.345	0.536	0.936
N-Acetyltryptophan	0.107	0.549	0.973
Maleic acid	0.313	0.554	0.864
Nicotinic acid	0.426	0.560	1.153
N-Acetyl-beta-D-mannosamine	0.632	0.564	1.296
6-Methylmercaptopurine	0.232	0.567	0.985
D-alanyl-D-alanine	0.530	0.575	1.153

22-Ketocholesterol	0.865	0.576	0.578
Benzoic acid	0.123	0.585	1.058
2-Monopalmitin	0.080	0.596	1.178
Thymine	0.404	0.627	0.811
Gluconic lactone	0.458	0.629	1.072
Adenine	0.421	0.651	1.095
L-dopa	0.245	0.670	0.916
2-Deoxyerythritol	0.256	0.677	0.949
Methyl-beta-D-galactopyranoside	0.526	0.679	1.132
Glutaraldehyde	0.055	0.679	1.000
Malonic acid	0.405	0.684	0.775
4-Methylbenzyl alcohol	0.457	0.692	1.061
Lyxose	0.326	0.703	0.897
2,4-Diaminobutyric acid	0.062	0.709	0.973
L-Threose	0.748	0.711	1.091
4-Androsten-11beta-ol-3,17-dione	0.412	0.727	0.943
Indolelactate	0.022	0.732	1.005
Lysine	0.168	0.738	1.057
Cis-gondoic acid	0.373	0.791	1.098
1-Monopalmitin	0.655	0.792	1.058
Threonine	0.318	0.809	1.103
Orotic acid	0.101	0.809	0.971
Phosphate	0.760	0.829	1.020
Uridine monophosphate	0.523	0.849	0.916
Arachidonic acid	0.011	0.860	1.038
Norvaline	0.328	0.887	0.928
Beta-Alanine	0.115	0.906	0.977
Proline	1.004	0.911	1.194
Ascorbate	0.319	0.922	1.012
Creatine	0.099	0.922	1.017
4',5-Dihyrroxy-7-methoxyisoflavone	0.614	0.949	1.325
Lactic acid	0.360	0.957	0.666
Thymidine 5'-monophosphate	0.002	0.980	1.000

Table S3. Metabolic profiles of metabolites in TM4 cells

Metabolite	VIP	P Value	Fold change
1,3-diaminopropane	0.173	0.773	0.955
1-Methylhydantoin	0.714	0.311	1.237
1-Monopalmitin	0.751	0.534	1.441
2,3-Dihydroxypyridine	1.342	0.217	0.902
2,4-Diaminobutyric acid	0.061	0.564	0.994
22-Ketocholesterol	0.600	0.629	0.660
2'-Deoxycytidine 5'-triphosphate	0.713	0.123	0.698
2-Deoxyerythritol	0.763	0.381	0.625
2-Deoxyuridine	0.044	0.256	0.913
2'-Hydroxyacetophenone	0.822	0.469	0.587
2-Hydroxypyridine	1.286	0.374	0.000
2-Ketoadipate	1.302	0.342	0.724
2-Ketobutyric acid	0.060	0.639	1.012
2-Monoolein	0.708	0.628	0.594
2-Monopalmitin	0.706	0.362	0.496
3 Beta-Hydroxy-5beta-pregnane-20-one	1.375	0.267	1.814
3,4-Dihydroxymandelic acid	0.753	0.325	0.651
3-Hydroxypyridine	0.083	0.789	1.018
3-Methylthiopropylamine	0.305	0.813	1.027
3-Ureidopropionate	1.809	0.050	1.341
4',5-Dihyrroxy-7-methoxyisoflavone	1.290	0.456	0.797
4-Acetamidobutyric acid	0.438	0.634	1.165
4-Aminobutyric acid	0.782	0.702	1.092
4-Androsten-11beta-ol-3,17-dione	1.386	0.054	1.288
4-Methylbenzyl alcohol	0.761	0.446	1.463
4-Vinylphenol dimer	0.505	0.453	1.204
5-Aminovaleric acid	0.402	0.776	0.840
5-Methoxytryptamine	0.250	0.178	1.169
5'-Methylthioadenosine	0.017	0.825	1.000
6-Methylmercaptopurine	1.574	0.145	0.460
7-Hydroxy-4-androstene-3,17-dione	0.572	0.628	0.516
8-Aminocaprylic acid	1.563	0.060	1.127
9-Fluorenone	0.496	0.560	0.656
Adenine	0.333	0.500	1.133
Adenosine	1.781	0.036	0.650
Alanine	0.041	0.782	0.984
Alpha-ketoglutaric acid	0.022	0.273	1.007
Aminomalonic acid	1.476	0.113	1.758
Ascorbate	0.875	0.392	0.189
Asparagine	1.362	0.140	1.704
Aspartic acid	0.046	0.943	1.012

Benzoic acid	1.470	0.363	0.708
Beta-Alanine	0.136	0.442	0.980
Beta-Mannosylglycerate	0.601	0.196	0.507
Biuret	0.445	0.686	1.232
Carnitine	0.116	0.601	1.037
Cis-gondoic acid	1.035	0.027	1.339
Citraconic acid	1.306	0.139	0.674
Citric acid	1.585	0.061	0.498
Citrulline	0.731	0.326	1.419
Conduritol b epoxide	0.251	0.553	1.043
Creatine	2.263	0.048	2.001
Cumic Acid	1.171	0.339	0.882
Cyclohexylsulfamic acid	0.894	0.148	3.541
Cycloleucine	0.864	0.233	1.182
Cytidine-monophosphate	0.307	0.625	1.264
Cytosin	0.016	0.776	0.994
D-(glycerol 1-phosphate)	0.907	0.175	0.700
D-alanyl-D-alanine	0.705	0.493	1.224
D-erythronolactone	0.796	0.186	2.282
D-Glyceric acid	0.051	0.948	0.992
Diglycerol	0.035	0.844	0.990
Dihydrotestosterone	0.754	0.312	1.473
Dioctyl phthalate	0.263	0.436	1.164
Dithioerythritol	0.409	0.674	0.950
dTMP	1.500	0.030	0.649
Elaidic acid	0.102	0.186	1.040
Ethanolamine	0.372	0.773	1.037
Farnesal	1.030	0.817	0.563
Fructose	0.739	0.201	0.933
Fumaric acid	0.118	0.853	0.978
Galactinol	0.533	0.781	0.822
Gluconic lactone	0.228	0.590	1.028
Glucose	0.966	0.332	0.655
Glutamic acid	1.057	0.202	1.209
Glutamine	2.424	0.007	2.495
Glutaraldehyde	0.229	0.781	0.973
Glutathione	1.122	0.276	1.228
Glycine	0.777	0.546	0.899
Glycocyamine	0.997	0.538	0.158
Guanine	0.053	0.611	0.991
Guanosine-5'-monophosphate	0.511	0.664	0.734
Heptadecanoic acid	0.584	0.223	1.182
Hydrocinnamic acid	1.422	0.095	1.229
Hydrocortisone	1.035	0.018	0.522

Hydroxylamine	2.040	0.043	1.317
Hypoxanthine	0.971	0.157	1.253
Indolelactate	1.551	0.044	0.545
Isoleucine	1.403	0.103	1.218
Lactic acid	1.942	0.072	1.607
Lactose	0.119	0.484	1.026
L-Allothreonine	0.571	0.658	1.037
L-cysteine	1.098	0.233	0.842
L-dopa	0.087	0.761	0.987
L-homoserine	0.344	0.544	1.082
L-Malic acid	0.412	0.385	0.867
L-Threose	1.534	0.183	0.713
Lysine	0.378	0.634	1.068
Lyxose	1.594	0.163	0.440
Maleic acid	0.365	0.150	0.896
Maleimide	0.344	0.779	0.957
Malonamide	0.463	0.725	0.794
Malonic acid	1.793	0.033	0.712
Methionine	0.859	0.447	1.106
Methyl heptadecanoate	1.300	0.213	0.540
Methyl Palmitoleate	0.332	0.485	1.114
Methyl Phosphate	0.257	0.589	0.957
Methyl-beta-D-galactopyranoside	1.786	0.089	2.090
Methylmalonic acid	0.270	0.687	0.857
Myo-inositol	0.128	0.661	0.956
Myristic Acid	1.092	0.153	0.611
N-(3-aminopropyl)-morpholine	0.213	0.305	1.051
N(epsilon)-Trimethyllysine	0.860	0.043	0.792
N-Acetyl-beta-D-mannosamine	0.126	0.273	0.980
N-Acetylisatin	0.236	0.820	0.788
N-Acetyltryptophan	0.143	0.609	0.974
N-alpha-Acetyl-L-ornithine	1.092	0.032	1.282
Naphthalene	2.004	0.030	1.303
N-Ethylglycine	0.779	0.548	0.914
Nicotinamide	1.092	0.108	0.812
Nicotinic acid	1.048	0.026	1.430
N-methylaniline	0.593	0.260	0.905
N-Methyl-DL-alanine	0.669	0.563	0.935
N-Methyl-L-glutamic acid	0.004	0.412	0.998
Noradrenaline	2.424	0.027	1.424
Norleucine	0.673	0.631	0.644
Norvaline	0.807	0.366	1.089
O-methylthreonine	0.376	0.026	0.873
O-Phosphorylethanolamine	0.105	0.223	0.961

O-Phosphoserine	1.077	0.064	0.812
Ornithine	1.560	0.143	1.931
Orotic acid	1.057	0.095	0.608
Oxalic acid	0.058	0.783	1.012
Oxoproline	1.220	0.216	1.134
Pantothenic acid	1.717	0.024	1.200
Pelargonic acid	0.425	0.670	0.879
Pentadecanoic acid	1.096	0.301	0.223
Phenylalanine	1.441	0.075	1.338
Phenylethylamine	0.538	0.629	0.950
Phosphate	1.046	0.456	1.101
Phosphomycin	0.232	0.669	1.100
Phthalic acid	1.105	0.310	0.811
Proline	0.732	0.193	1.164
Prostaglandin E2	0.014	0.296	0.972
Putrescine	0.684	0.373	0.535
Pyrophosphate	0.918	0.415	0.561
Ribitol	0.780	0.333	1.117
Serine	0.031	0.877	1.022
Shikimic acid	0.344	0.097	1.121
Sorbitol	1.506	0.063	1.544
Sorbose	0.729	0.313	1.363
Spermidine	2.039	0.036	0.367
Synephrine	0.322	0.355	1.069
Taurine	0.900	0.632	0.889
Threitol	1.469	0.192	0.705
Thymidine	1.770	0.020	0.356
Thymine	0.179	0.655	1.074
Thymol	1.043	0.402	0.835
Tricetin	0.269	0.904	0.940
Tyrosine	0.356	0.258	0.947
Uracil	2.048	0.019	2.074
Uridine monophosphate	2.546	0.272	2.560
Valine	0.860	0.485	1.118
Zymosterol	0.581	0.481	1.288
